# Adverse Effects of Excessive Folic Acid Consumption and Its Implications for Individuals With the Methylenetetrahydrofolate Reductase C677T Genotype

**DOI:** 10.7759/cureus.79374

**Published:** 2025-02-20

**Authors:** Jessica Hecker, Rhett Layton, Robert W Parker

**Affiliations:** 1 Emergency Medicine, Alabama College of Osteopathic Medicine, Dothan, USA; 2 Pharmacology, Alabama College of Osteopathic Medicine, Dothan, USA

**Keywords:** folate, folic acid, mthf, mthfr, obstetrics, spina bifida

## Abstract

This scoping review examines the risks of high folic acid supplementation in expectant mothers, particularly those with the methylenetetrahydrofolate reductase (MTHFR) C677T gene polymorphism. While folic acid is essential for DNA synthesis and preventing neural tube defects, excessive intake may pose health risks, especially for individuals with the MTHFR variant. These individuals have a reduced ability to process folic acid, leading to the accumulation of unmetabolized folic acid (UMFA), which is associated with vitamin B12 deficiency, cognitive and psychiatric issues, and adverse pregnancy outcomes. This review emphasizes the importance of monitoring folic acid intake and explores alternative supplements such as 5-MTHF or 5-FTHF, which may mitigate these risks by bypassing MTHFR conversion. Personalized nutrition, considering genetic variations, is crucial for optimizing health and minimizing potential harm.

## Introduction and background

Folate

Folate is a vitamin naturally found in foods like leafy greens, fruits, beans, and whole grains; it is important for quality health, especially during pregnancy [1]. Folate helps the body make methionine, an essential substance for producing a compound called S-adenosyl-methionine (SAM), which is involved in processes such as DNA methylation [1]. DNA methylation is one way the body controls which genes are turned on or off, helping regulate the production of certain proteins. When this process does not work properly, it can cause harmful buildups in the body.

How the cell uses folate largely depends on a gene called the methylenetetrahydrofolate reductase (MTHFR) gene. MTHFR helps convert folate into a form that can be used to convert homocysteine into methionine, both of which are found in the body. If homocysteine builds up because of a decreased ability to be converted into methionine, it can block the production of important brain chemicals like serotonin and norepinephrine, which are linked to mood and depression [2]. When patients have a mutation in the MTHFR gene, they have a decreased ability to process folate. Screening for this genetic mutation would be beneficial for these individuals to avoid an imbalance of this aforementioned imperative vitamin. 

During pregnancy, the body requires more folate due to its effect on preventing birth defects, such as neural tube defects (NTDs), which occur in the first few weeks of pregnancy [3]. NTDs, such as spina bifida and anencephaly, affect the spine and nervous system, leading to serious health problems for the baby [4]. To help avoid these issues, pregnant women need to have an adequate amount of folate in their system.

The average individual primarily controls their folate intake through dietary means. However, dietary folate is less stable and not as easily absorbed as folic acid, the synthetic version that is found in supplements [1]. Folic acid supplementation is the most common way for individuals with increased needs, such as during pregnancy, to boost their daily intake. Despite the importance of folate during pregnancy, doctors infrequently test folate levels unless there is a specific reason, such as previous unsuccessful pregnancies. The American College of Obstetricians and Gynecologists [5] recommends that pregnant individuals take a daily prenatal vitamin with at least 400 µg of folic acid, starting one month before getting pregnant and continuing through the first trimester, to lower the risk of birth defects. Aside from pregnancy, folate supplementation is generally not recommended for most individuals. In fact, it is paramount that folate levels stay within normal limits to avoid serious adverse effects from excess folate in the bloodstream.

Folic acid

Folic acid, the synthetic version of folate, is often used in food fortification and daily supplements due to its cost-effectiveness and stability [6]. Folic acid is essential for human health, as it plays a crucial role in the synthesis of DNA, RNA, and proteins. It also supports the formation of red blood cells and is vital for brain function [7]. Along with vitamin B12, as mentioned previously, folic acid is essential in the metabolism of homocysteine. When deficient, homocysteine levels rise, increasing the risk of cardiovascular diseases [6] and other adverse physiological effects in the body which extends past the scope of this current project. The full extent of homocysteine’s direct effects on the body requires further research. For the present study, homocysteine will be discussed in terms of its relationship to folic acid levels, and how these varying levels may influence downstream occurrences in the body.

**Figure 1:**
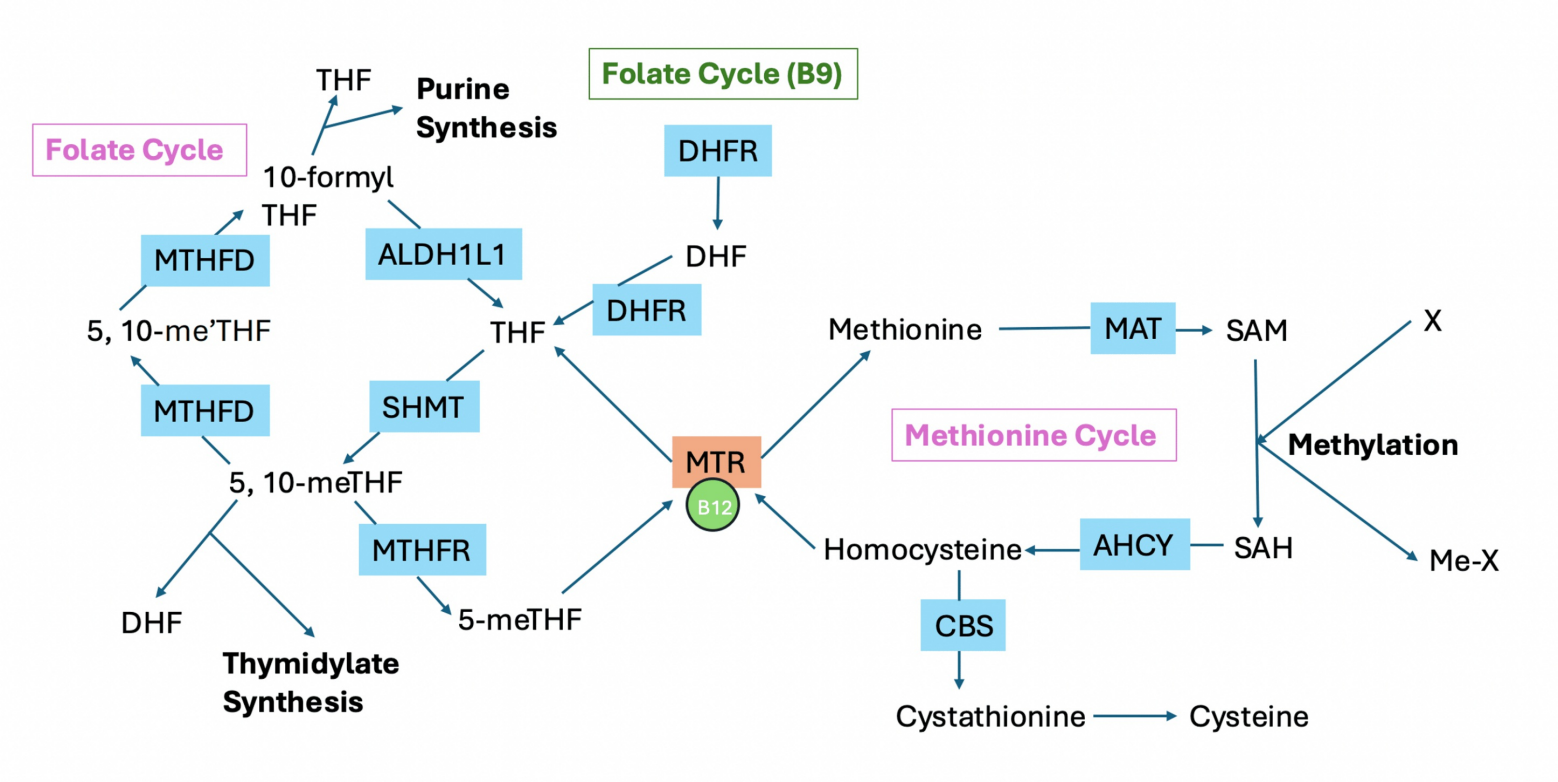
Schematic of one-carbon metabolism. Note: This figure includes the folate and methionine cycles. X represents a methylation target. MTHFD: methylenetetrahydrofolate dehydrogenase, ALDH1L1: aldehyde dehydrogenase 1 family members L1, SHMT: serine hydroxymethyltransferase, MTHFR: methylenetetrahydrofolate reductase, DHFR: dihydrofolate reductase, MTR: methionine synthase reductase, MAT: methionine synthase, AHCY: adenosylhomocysteinase, CBS: cystathionine β-synthase, B9: vitamin B9, B12: vitamin B12, DHF: dihydrofolate, THF: tetrahydrofolate, 5-me-THF=5-methyl-THF, 5-me+-THF=5-methylene-THF, SAM: S-adenosyl-methionine, SAH: S-adenosyl-homocysteine. Image credit:  Created by authors Hecker and Layton using Microsoft PowerPoint (Microsoft Corp., Redmond, WA, United States).

Folic acid, when consumed, exists in an inactive form that must be converted by the liver into its active state, 5-methyltetrahydrofolate (5-MTHF), which is necessary for various metabolic functions. Very few adverse effects of excess natural folate with 5-MTHF supplementation have been documented, and for this reason, they do not apply to concerns such as the accumulation of unmetabolized folic acid (UMFA) in the blood. UMFA in the bloodstream has been associated with adverse health effects, such as cognitive impairment, cleft lip, and autism [8]. Figure 1 illustrates the mechanism by which folic acid is converted to its active form (5-MTHF) and the subsequent conversion of homocysteine to methionine. This conversion leads to the production of S-adenosyl-methionine (SAM), which is critical for DNA methylation.

A common genetic variation that deserves attention when discussing the upper limits of folic acid is the MTHFR gene, specifically the C677T variant, which can lead to higher levels of unmetabolized folic acid in the blood. This buildup has been linked to health issues such as depression, bipolar disorder, schizophrenia, and heart disease [9]. The MTHFR gene is responsible for converting folic acid into its active form (5-MTHF), and when this conversion does not happen properly, folic acid can accumulate in the body. Individuals with this genetic mutation are at increased risk of having elevated folic acid levels in their blood, which can lead to the aforementioned adverse effects. According to a study by de Góes Soligo et al. [9], the MTHFR C677T mutation was found in 49.2% of fertile women and 58.5% of infertile women. This mutation impairs the conversion of folic acid to L-methylfolate, resulting in folic acid buildup and potentially elevated homocysteine levels, which can have adverse effects as mentioned previously. A significant aspect of this review is understanding not only the adverse effects of the upper limits of folic acid in expectant mothers but also the increased risk of these adverse effects in mothers with the MTHFR C677T mutation. 

Folic acid dosage

According to WebMD, the Centers for Disease Control and Prevention (CDC) recommends women start taking folic acid daily for at least a month before becoming pregnant and continue taking it throughout pregnancy. Additionally, the CDC advises that all women of childbearing age take folic acid daily [7]. To prevent birth defects, in 1998, the Institute of Medicine (IOM) recommended that pregnant women consume 400 μg of folic acid daily from fortified foods and supplements [6], while the upper limit for folic acid intake was set at 1000 μg per day in the same year. Women of childbearing age are strongly advised to consume at least 400 μg of folic acid daily since the neural tube develops within 28 days following conception [6]. During this period, the fetus is highly vulnerable to developing birth abnormalities; however, the use of folic acid has been proven to reduce these risks.

Folic acid is readily available from both over-the-counter (OTC) preparations and prescription medications. OTC supplements commonly contain 400-800 μg of folic acid, while prescription forms usually provide 1 mg. This availability makes it easier for women of reproductive age to meet their daily requirements [7]. In both the United States and Canada, recent reports have shown that women of childbearing age often exceed the recommended folic acid intake of 1 mg daily because of mandatory fortification combined with supplemental use [10]. The impact of maternal over-supplementation for purposes of NTDs is largely under-documented. Several studies have been investigated to determine the average intake of childbearing and expectant women. In Canada, Ray [11] conducted a study to examine the number of women from a cohort that had suboptimal folate status. The data from this clinical laboratory study included 1035 individuals, with 235 women between the ages of 15 and 45. Measures of RBC folate revealed that 7% of women had suboptimal folate levels. These findings suggest that women in this sample were receiving adequate amounts of folate to prevent NTDs. While the sample size was rather small and these were not expectant mothers, it provides a foundational assessment of the folate needs of women in Canada.

## Review

Detriments of upper limits of folic acid

Folic acid supplementation, known for its role in preventing NTDs in pregnant women, led to the introduction of folic acid fortification programs by the Canadian and US governments in 1998 to increase folate intake [6,12]. Recently, mandatory folic acid fortification combined with the use of folic acid supplements has been scrutinized, as several studies have reported that the chemically synthesized form of folates can cause adverse health effects [13-15]. This emphasizes the need for further discussion on the safe use of folic acid and potential adjustments to current medical practices.

While folic acid is essential for DNA synthesis, repair, and methylation, excess intake raises concerns, particularly in its synthetic form [16]. High doses of folic acid, above 1 mg/day, can mask the symptoms of vitamin B12 deficiency, which can lead to serious neurological damage, especially in patients with megaloblastic anemia [7]. Additionally, folate and its derivatives, including folic acid, are sensitive to UV radiation, which can degrade folate in the blood and skin. Studies suggest that UV exposure may contribute to folate degradation, further complicating the balance of folic acid intake [7,8].

Like any drug, folic acid has the potential for possible side effects. Murray et al. [17] present several adverse effects that can stem from elevated levels of folic acid in the blood. It is mentioned that high levels of folic acid can mask the symptoms of anemia caused by low vitamin B12, which can result in serious brain and nerve damage if left untreated. Additionally unmetabolized folic acid can cause issues such as DNA damage, leading to an increased risk of cancer and digestive complications. Additionally, high levels of folic acid, especially above the recommended limit, have been shown to decrease the activity of natural killer (NK) cells, which are important for our body’s immune system. This is particularly concerning for older women, as lower NK cell activity can make it harder for the body to fight off cancer. Excess folic acid has also been connected to heart disease, autism, and birth defects like cleft lip. These issues highlight the importance of properly managing folic acid intake to prevent health issues while still supporting the body’s essential functions. As more side effects come to light, the need for further research is necessary to confirm complications of excess folic acid.

According to WebMD, the CDC recommends women start taking folic acid daily for at least a month before becoming pregnant and continue taking it throughout pregnancy. Additionally, the CDC advises that all women of childbearing age take folic acid daily [8]. To prevent birth defects, in 1998, the IOM recommended that pregnant women consume 400 μg of folic acid daily from fortified foods and supplements [6], while the upper limit for folic acid intake was set at 1000 μg per day in the same year. Women of childbearing age are strongly advised to consume at least 400 μg of folic acid daily since the neural tube develops within 28 days following conception [6]. During this period, the fetus is highly vulnerable to developing birth abnormalities; however, the use of folic acid has been proven to reduce these risks.

There are two main ways in which a buildup of folic acid in the blood can occur: the first is through excess intake beyond the body’s needs, and the second is the inability to fully metabolize folic acid due to genetic mutations, such as those affecting the MTHFR gene. While folic acid is crucial for growth and development, particularly during pregnancy, excessive levels can lead to adverse effects, which highlights the need for a balanced intake approach [10]. Safe supplementation, alongside personalized nutrition based on genetic variations, is important for long-term health and the well-being of both the mothers and their children.

In a study by Hoyo et al. [14], 539 mothers in the United States were interviewed about their folate supplementation before and during pregnancy. Approximately 12% reported taking folic acid above the upper limit of 1000 μg per day, with older, married, White, privately insured women with higher education being more likely to exceed the limit. This study raises concerns about the varying levels of folic acid intake and suggests that further research is needed to examine the influence of factors like ethnicity, background, and income on supplementation habits.

A similar study in Spain by Valera-Gran et al. [18] found that 29% of pregnant women exceeded the recommended daily dose of folic acid from preconception to the third month of pregnancy, and 17% remained above the limit from the fourth to seventh months. This study examined both OTC and prescription folic acid intake and included dietary sources, highlighting the need for more comprehensive data on folic acid intake across different populations.

Research performed by Fardous et al. [19] displayed the outcomes of excess folic acid and how it can have a negative effect on health. They found that high levels of folic acid can increase the risk of gestational diabetes during pregnancy, as well as raise the chances of developing colorectal cancer, breast cancer, and hormone-related disorders. The study emphasizes the need to carefully monitor both the amount and form of folic acid being consumed, to avoid these risks.

A concern with the over-supplementation of folic acid is not only its effects on the mother but also on the fetus. There are many potential adverse effects of maternal intake above the upper limit, including low psychomotor scores, autism, decreased verbal memory, language delay, reduced embryonic size, and insulin resistance [11]. As awareness of the risks associated with low folate levels in fetuses grows, there may be a shift toward greater use of OTC vitamins, including folic acid. A push for more involvement in the management of folic acid supplementation is needed to circumvent these potential adverse effects.

Postpartum depression (PPD) is a clinically significant aspect of a mother's pregnancy journey. PPD most commonly occurs within six weeks after childbirth and occurs in approximately 6.5%-20% of postpartum women in the United States [20]. While the etiology of PPD is not well understood, the relation of folate metabolism pathways is of interest. 

A case-control study by James et al. [21] in the United States examined the relationship between folate supplementation and the development of autism spectrum disorder (ASD). They compared plasma levels of several metabolites involved in folate and methionine metabolism in children with ASD and control children ages 6-8 years old. Children with ASD had lower plasma levels of methionine, SAM, and homocysteine, with higher levels of S-adenosyl-homocysteine and adenosine. The findings suggest that impairments in the methylation process often affect children with ASD. In another study by Raghavan et al. [15], maternal folate supplementation was investigated through a longitudinal study. This data discovered that high levels of plasma vitamin B12 (>600 pmol/L) and folate (>59 nmol/L) were associated with a higher risk of the fetus developing ASD. This study highlighted the increased risk of ASD in expectant mothers with elevated levels of both vitamin B12 and folate. It also considered the mother’s MTHFR genotype, essential in the metabolism of folate. As awareness of both the benefits and potential risks of folic acid supplementation grows, the need for careful management during pregnancy rises. While adequate folate intake is crucial in preventing NTDs, excessive supplementation may pose risks to both maternal and fetal health. A balanced approach that monitors appropriate folate levels is crucial to prioritize the well-being of both the mother and the child.

Without routine testing for folic acid levels, particularly in the absence of a history of NTDs, OB/GYNs may not identify patients who exceed the upper limit of folic acid intake. Folic acid remains an essential tool in preventing NTDs, but medical professionals must continue to assess its use to ensure patient safety. The widespread use of folic acid supplements necessitates ongoing research to ensure safe and effective use. Studies must explore intake levels, metabolic pathways, and the potential health risks associated with folic acid.

Detriments of psychological effects

A mutation in the MTHFR gene not only affects folate metabolism leading to its own mass of complications, but it can be related to the development of psychiatric disorders in the mother. A meta-analysis by Zhang et al. [2] investigated the correlation between severe psychological disorders (major depression, bipolar disorder, and schizophrenia) and MTHFR single-nucleotide polymorphisms such as C677T and A1298C. This study concluded that MTHFR C677T polymorphism is significantly related to schizophrenia and major depression, with an increased risk of bipolar disorder in recessive models, revealing the significant role MTHFR may play in the common pathogenesis of mental illness. 

Therefore, women with the MTHFR C677T genotype become more affected by rising demands of folate and, in turn, suffer more adverse effects. A study by Morris et al. [22] examined the relationship between MTHFR genotype variants and depression in postpartum women. The data presumed that low folate (therefore high homocysteine levels) is associated with various forms of psychopathology. However, this inverse relationship may only be significant in women with the MTHFR C677T allele, concerning manic and depressive symptoms. This study aligns with the hypothesis that low folate alone can lead to depressive symptoms and can be further exacerbated by an MTHFR mutation. 

The exploration into the MTHFR C677T gene, which plays a crucial role in folic acid metabolism, provides important context for understanding the relationship between health effects and folic acid consumption. Variations in the MTHFR gene demonstrate the importance of proper folic acid supplementation. It is crucial that these individuals do not exceed the recommended dose, to avoid adverse effects.

Alternative options to folic acid

Natural folate is mainly in the form of 5-MTHF and a lesser form of 5-formyl-tetrahydrofolate (5-FTHF) [20]. These two forms are biologically active and do not require activation by dihydrofolate reductase (DHFR), unlike folic acid. 

5-MTHF is the active form of folate that can be absorbed through the diet or generated in the liver from folic acid. This scoping review report advocates for the increased use of 5-MTHF rather than folic acid. A primary reason for this recommendation is that 5-MTHF does not require activation by the DHFR enzyme. This implies that 5-MTHF does not rely on DHFR, an enzyme that can be genetically mutated; therefore, intake of 5-MTHF cannot lead to high accumulation levels in the blood [23]. MTHFR and DHFR are both enzymes that play crucial roles in the metabolism of folic acid. A common variant is MTHFR C677T, seen most often in individuals of European descent [24]. Using 5-MTHF in place of folic acid can mitigate the aforementioned adverse effects as it is not at risk of harmful accumulation levels.

5-MTHF is less reactive toward DHFR inhibitors, such as methotrexate, which results in decreased masking of hematological symptoms associated with vitamin B12 deficiency. Therefore, consideration of 5-MTHF as a replacement in patients with megaloblastic anemia should be further reviewed. 5-MTHF should also be considered as the primary supplement for pregnant women due to its stability in UV-A radiation and visible light. Therefore, in individuals who are consistently exposed to sunlight, replacing folic acid with 5-MTHF can ensure greater stability in blood serum folate levels. These adverse effects have not been observed with the upper limit intake of 5-MTHF, due to its feedback loop and, therefore, the inability to result in excess folate levels in tissues. 

However, the benefits of using 5-MTHF rather than folic acid come at a cost. Folic acid is more heat-stable than the active vitamin and is significantly less expensive, making it the generally preferred form for production, sales, and therapeutic use [15]. As research progresses, one area of potential action would be reducing the cost of 5-MTHF, to provide high-risk patients with a safer alternative to folic acid and to advocate for the routine screening of folic acid levels and search for additional ways to make 5-MTHF more available to the general public for those more at risk to the adverse effects of the upper limits of folic acid. 

Taking into consideration the benefits of 5-MTHF over folic acid, the findings of this scoping review support the change in supplementation and prescribing practices. As the data on women ingesting the upper limits of folic acid continues to grow, further research should focus on safer and more comprehensive alternatives for both maternal health and the developing fetus.

The medical use of folic acid to prevent NTDs in pregnant women has been a crucial public health strategy in Canada and the United States since the late 1990s. However, recent scrutiny has raised concerns about potential adverse effects associated with high intake of chemically synthesized folic acid. This scoping review examines current medical practices involving folic acid, explores its adverse effects, and discusses potential alternatives, particularly in light of the MTHFR C677T genotype.

Studies indicate that many women of childbearing age exceed recommended folic acid intake levels due to mandatory fortification and supplemental use. This overconsumption raises concerns, as it may lead to unintended health consequences, particularly during pregnancy. Excessive folic acid intake has been linked to adverse effects such as increased risk of autism, developmental delays, and insulin resistance in fetuses. Moreover, research suggests that a significant portion of pregnant women surpass the upper limits of folic acid intake, underscoring the need for better management and monitoring of supplementation practices.

Genetic variations, such as the MTHFR C677T mutation, further complicate folic acid metabolism. This mutation, prevalent among both fertile and infertile women, affects the body's ability to convert folic acid into its active form, potentially leading to elevated homocysteine levels and associated health risks. Personalized supplementation approaches that consider genetic factors like MTHFR status are therefore crucial in optimizing folate intake during pregnancy.

The case for adopting 5-MTHF over folic acid is compelling. Unlike folic acid, 5-MTHF is the bioactive form of folate and does not require enzymatic activation by DHFR. This distinction is critical as it mitigates concerns about excessive accumulation in the bloodstream and reduces risks associated with masking vitamin B12 deficiency symptoms.

Although folic acid remains crucial in preventing NTDs, its synthetic nature and potential adverse effects, particularly in individuals with the MTHFR C677T genotype, warrant reconsideration of its use. The adoption of 5-MTHF as a primary supplement offers a promising solution, addressing concerns related to overconsumption and genetic variability in folate metabolism. Continued research and advocacy for safer supplementation practices are essential to ensure the health and well-being of both maternal women and developing fetuses. This shift toward 5-MTHF could lead to improved maternal and child health outcomes, thereby advancing public health initiatives aimed at preventing birth defects and promoting overall well-being.

## Conclusions

The medical use of folic acid to prevent NTDs in pregnant women has been a cornerstone of public health strategies in Canada and the United States since the late 1990s. However, increasing scrutiny has raised concerns about the potential adverse effects of excessive intake of chemically synthesized folic acid, particularly as many women of childbearing age now exceed recommended intake levels due to mandatory fortification and supplemental use. Overconsumption of folic acid has been linked to a range of health issues, including an increased risk of autism, developmental delays, and insulin resistance in fetuses, highlighting the need for better management and monitoring of supplementation practices. Compounding this issue, genetic variations such as the MTHFR C677T mutation, which affects the body's ability to convert folic acid into its active form, further complicate folate metabolism and may elevate homocysteine levels, posing additional health risks. Personalized approaches to supplementation, taking into account genetic factors like MTHFR status, are therefore essential to optimizing folate intake during pregnancy. In this context, the use of 5-MTHF, the bioactive form of folate, emerges as a promising alternative to folic acid. Unlike folic acid, 5-MTHF does not require enzymatic activation and bypasses concerns related to excessive folic acid accumulation and the masking of vitamin B12 deficiency symptoms. While folic acid remains critical for preventing NTDs, its synthetic nature and potential adverse effects, particularly in individuals with the MTHFR C677T genotype, suggest a need for reconsideration of its use. Transitioning to 5-MTHF as a primary supplement could address these concerns, offering a safer and more effective solution tailored to individual needs and genetic profiles. Continued research and advocacy for safer supplementation practices are vital to safeguarding maternal and fetal health, ensuring improved outcomes, and advancing public health initiatives aimed at preventing birth defects and promoting overall well-being.
